# Supplementary material for: Mitochondrial ClpX activates an essential biosynthetic enzyme through partial unfolding
Source: eLife. 2020 Feb 24;9:e54387. doi: 10.7554/eLife.54387 (PMC7077987; doi:10.7554/eLife.54387)
Supplement: Supplementary file 2. — All strains were made in w303 mat a background (MATa ade2-1 leu2-3 ura3 trp1-1 his3-11,15 can1-100 GAL psi+). [file elife-54387-supp2.docx]

**Supplementary File 2**

*S. cerevisiae* strains used in this work. All strains were made in w303 mat a background (*MATa ade2-1 leu2-3 ura3 trp1-1 his3-11,15 can1-100 GAL psi+*)

| **strain** | **genotype** |
| --- | --- |
| JKY194 | *hem1Δ::6HA*NAT* |
| JKY195 | *hem1Δ::6HA*NAT mcx1Δ::KAN* |
| JKY196 | *hem1(F71A-Y73A)-3MYC*TRP1* |
| JKY197 | *hem1(F71A-Y73A)-3MYC*TRP1 mcx1Δ::KAN* |
| JKY198 | *HEM1-3MYC*TRP1* |
| JKY199 | *HEM1-3MYC*TRP1 mcx1Δ::KAN* |
| JKY220 | *hem1-MYC-7HIS*KAN* |
| JKY229 | *hem1-F71A-3MYC*TRP1* |
| JKY230 | *hem1-Y73A-3MYC*TRP1* |
| JKY231 | *hem1-F71A-3MYC*TRP1, mcx1Δ::KAN* |
| JKY232 | *hem1-Y73A-3MYC*TRP1, mcx1Δ::KAN* |
| JKY236 | *hem1-Y274A-3MYC*TRP1* |
| JKY237 | *hem1-Y274A-3MYC*TRP1, mcx1Δ::KAN* |
| JKY238 | *hem1-F71A-Y274A-3MYC*TRP1* |
| JKY239 | *hem1-F71A-Y274A-3MYC*TRP1, mcx1Δ::KAN* |
